# Text messaging to improve retention in hypertension care in Bangladesh

**DOI:** 10.1038/s41371-024-00942-1

**Published:** 2024-08-24

**Authors:** Shamim Jubayer, Jubaida Akhtar, Ahmad Khairul Abrar, Md. Noor Nabi Sayem, Shahinul Islam, Khondoker Ehsanul Amin, Muhtamim Fuwad Nahid, Mahfuzur Rahman Bhuiyan, Mohammad Abdullah Al Mamun, Abdul Alim, Mohammad Robed Amin, Daniel Burka, Prabhanshu Gupta, Di Zhao, Kunihiro Matsushita, Andrew E. Moran, Sohel Reza Choudhury, Reena Gupta

**Affiliations:** 1 National Heart Foundation Hospital & Research Institute, Dhaka, Bangladesh; 2 Directorate General of Health Services, Ministry of Health and Family Welfare, Dhaka, Bangladesh; 3 Resolve to Save Lives, New York, NY USA; 4 Nilenso Software, Bengaluru, Karnataka India; 5 Johns Hopkins University, Baltimore, MD USA; 6 Columbia University Irving Medical Center, New York, NY USA; 7 University of California San Francisco, San Francisco, CA USA

**Keywords:** Hypertension, Disease prevention

## Abstract

Visit non-attendance is a common barrier to hypertension control in low and middle-income countries (LMICs). We aimed to evaluate the effectiveness of mobile text messaging in improving visit attendance among patients with hypertension in primary healthcare facilities in Bangladesh. A randomized A/B testing study was conducted with two patient groups: (1) patients regularly attending visits (regular patients) and (2) patients overdue for their follow-up clinic visit (overdue patients). Regular patients were randomized into three groups: a cascade of three text reminders, a single text reminder, or no text reminder. Overdue patients were randomized into two groups: a single text reminder or no text reminder. 20,072 regular patients and 12,708 overdue patients were enrolled. Among regular patients, visit attendance was significantly higher in the cascade reminder group and the single reminder group compared to the no reminder group (78.2% and 76.6% vs. 74.8%, *p* < 0.001 and 0.027, respectively). Among overdue patients, the single reminder group had a 5.8% higher visit attendance compared to the no reminder group (26.5% vs. 20.7%, *p* < 0.001). The results remained consistent in multivariable analysis; adjusted prevalence ratio (PR) was 1.04 (95% CI 1.02–1.06) for the cascade reminder group and 1.02 (95% CI 1.00–1.05) for the single reminder group among regular patients. The adjusted PR for the single reminder group vs. the no reminder group among overdue patients was 1.23 (95% CI 1.15–1.33). Text message reminders are an effective strategy for improving retention of patients in hypertension treatment in LMICs, especially for patients overdue to care.

## Introduction

Hypertension is the leading cause of preventable death worldwide, accounting for 10.7 million deaths annually, the majority of which occur in low- and middle-income countries (LMICs) [1]. Less than 15% of people with hypertension worldwide have controlled blood pressure (BP) [2]. Bangladesh has experienced a rapid increase in hypertension [3]. According to the Bangladesh National STEPS Survey of 2018, the prevalence of hypertension was 21% while the control rate was only 14% [4]. Aiming to increase the rate of hypertension control, the Bangladesh Hypertension Control Initiative (BHCI) program was started in 2018 at primary health care facilities across the country.

Loss to follow-up often occurs in 50% or more of patients with hypertension and is a significant barrier for controlling hypertension in LMICs [5–9]. For example, by the end of 2021, 44% of registered patients in the BHCI program had not visited the clinic for three months or more. In the BHCI, patients are given 30-day prescriptions and are expected to attend clinic visits every 30 days. Failure to attend follow-up visits is associated with adverse health outcomes [10, 11]. Effective strategies to reduce visit non-attendance and thereby retain treated patients in care are urgently needed to improve hypertension control in Bangladesh, as well as globally.

There is accumulating evidence about the potential for SMS-based messaging reminders to improve patient attendance rates at clinics in high-income countries (HICs) [12]. Evidence-based behavioral economic theory has shown that strategic text messaging can encourage patients to attend healthcare appointments [13]. In Bangladesh, cell phones are widely used [14]. Due to low cost, quick delivery, and minimal intrusiveness (e.g., compared to phone reminders), text messaging is an attractive strategy in health care services [15–17]. Several studies have found that text messages can improve medication adherence and reduce the rate of failure to attend scheduled appointments [18, 19]. However, few studies have been conducted to evaluate the effectiveness of text messages for improving visit attendance for hypertension in LMICs. An A/B testing study was carried out to evaluate the effectiveness of SMS messaging to improve patients’ clinic visit attendance for hypertension treatment in Bangladesh.

## Methods

### Study design and setting

The study is a randomized A/B test conducted in Bangladesh between December 2021 to January 2022. A/B tests are rapid-cycle randomized tests to compare two or more variants of a digital intervention. The study was conducted as part of BHCI, which was launched in 2018 to strengthen hypertension detection, treatment, and follow-up in primary health care facilities in collaboration among the National Heart Foundation of Bangladesh, the Bangladesh Ministry of Health and Family Welfare, and Resolve to Save Lives. The program is implemented in 182 Upazila Health Complexes (UHCs), 50-bed primary health centers that each serve a subdistrict (Upazila) population of 200,000 to 400,000, in seven districts of Bangladesh. The program involves implementation of an adapted World Health Organization HEARTS technical package which includes a simplified treatment protocol, team-based care, regular supply of free medications by the government, patient-centered approaches to care delivery, and implementation of the Simple application software. The Simple application is a mobile-based digital information system for managing hypertension [20, 21]. Patients receive 30 days of free antihypertensive medications on-site at each visit and are scheduled for return visits every 30 days.

### Participants and randomization

The study included adults 18 years and older who were diagnosed with hypertension and registered in the BHCI through the Simple application from April 2019 to November 2021. Patients with no valid cell phone number in the Simple application were not eligible for the study.

The A/B text messaging reminder study involved two patient groups: (1) patients with an upcoming appointment in the next 30 days, indicating regular participation in BHCI (regular patients) and (2) patients who were overdue for their scheduled appointment and last visited 35–365 days prior to randomization (overdue patients). Regular patients were randomized via a randomization program to one of three arms: (1) A cascade of three text message reminders sent the day prior, the day of, and three days after the scheduled appointment (cascade reminder group), (2) One text message reminder sent one day prior to the scheduled appointment (single reminder group), and (3) No reminder group which did not involve any text message reminder. Overdue patients were randomized to one of two arms: (1) One text message reminder sent at the time of study enrollment (single reminder group) and (2) No reminder group which did not involve any text message reminder. There was no cascade arm for overdue patients since there was no scheduled appointment to trigger cascade messages. Intervention messages were personalized by including the name of the participant and assigned healthcare facility (Table 1). All SMSs were sent in the local language (Bangla).

**Table 1:** Text message reminders for regular and overdue patients.

1st SMS	<Patient Name > , please visit <Facility> on <Date> for a BP measure and medicines.
2nd SMS	We are expecting you today. <Patient Name > , please visit <Facility> for a BP measure and medicines
3rd SMS	You are late for your BP medicines. <Patient Name > , please visit <Facility> as soon as possible for a BP measure and medicines.

^*^1st SMS was sent as a single reminder in both regular study and overdue study. Also, it was the 1st SMS for three cascade messages.

### Measurements and data collection

The primary outcome was visit attendance, defined as the proportion of patients who had a recorded visit in the Simple app within 15 days of the scheduled appointment (regular patients) or within 15 days of study enrollment (overdue patients).

Baseline information collected on sociodemographic variables included age, gender, geographic location (district); assigned healthcare facility; history of diabetes, kidney disease, heart attack or stroke; baseline BP; history of prior missed visits (defined as ≥ 1 three-month period without a visit in the past 12 months), and duration of care in BHCI program.

The visit data, and sociodemographic characteristics were recorded directly into the Simple application by nurses at the UHC facilities. Simple application data is directly uploaded to a secure cloud-based server (Amazon server) on a daily basis. No individual patient identifier was shared with the Simple application development team or study investigators. Electronic SMS data were collected electronically and uploaded to the secure server.

### Statistical analysis

A sample size of 15,500 was estimated to provide a minimal detectable difference of 2.5% in visit attendance between groups, assuming type I error of 5%, power of 80%, and baseline follow-up rate of 75%.

Descriptive analyses were performed for all the study variables and characteristics of the patients were compared between each randomized messaging group. Normally distributed continuous variables were presented as mean and standard deviation, and skewed continuous variables were presented as median and interquartile interval. Categorical variables were presented as count and percentage. Differences of the mean comparing participants in different messaging groups of normally distributed continuous variables were evaluated using *t*-tests. -Chi-square test was used to compare the difference of proportion across the groups. Prevalence ratios of follow-up visits with 95% confidence intervals associated with the cascade reminder group and single reminder group vs. no reminder group were calculated using marginal adjusted probabilities. Multivariable logistic regression analyses were used to compare visit attendance across the different message groups adjusting for age, gender, geographic location, history of diabetes, kidney disease, heart attack and stroke, baseline BP, history of missed visits, and duration in care.

The primary analysis excluded patients with duplicate records or those who were registered in error, had inadvertently moved appointments, or failed SMS messages. The mobile app user interface was set up intentionally to prompt healthcare workers to enter future appointment dates. However, as a result, healthcare workers were inadvertently able to move appointment dates any time a patient record was accessed in the Simple application. Therefore, if an appointment was moved during the study period without an associated visit, this was identified as an ‘inadvertent moved appointment’ and the patient was excluded due to unclear expected follow-up date. Secondary analysis was performed to include patients that were excluded due to duplicate or incorrect records, moved appointments, or failed SMS messages.

To explore the potential modifications in the effect of messages on visit attendance by specific message groups, we included two-way interaction terms of intervention with age groups ( < median vs. ≥ median), gender, history of missed visit, duration in care, time to last visit, and history of comorbidities (diabetes, kidney disease, heart attack, or stroke). Statistical interaction was assessed using Wald test. Statistical analyses were performed using Stata version 17.0 (StataCorp, College Station, Texas 77845 USA).

### Ethics

Ethical approval for the study was obtained from the Institutional Review Boards of the National Heart Foundation Hospital and Research Institute (NHF&RI), Bangladesh (Reference number: N.H.F.H.& R.I/4/14-7/Ad./860) and Vital Strategies (Reference number: IRB00010793). The study was determined to be exempt human subjects research as an initiative to improve usual care and not a clinical trial and not registered as such. The study received a waiver for individual informed consent, as it was determined not to confer additional risk to participants compared to usual care where some patients routinely receive text messages in BHCI program facilities. Text messages are sent intermittently and at some facilities; however, text messages are not standard of care in most primary care facilities in Bangladesh justifying a control group to assess effectiveness. Additionally, current national and regional guidelines do not recommend text messaging as part of standard of care for individuals living with hypertension in Bangladesh. Informed consent for receiving electronic mobile-phone messages and storing data for program improvement was obtained for all patients during BHCI program registration in the Simple application. Obtaining additional consent for this specific study would cause significant logistic barriers and compromise study results. This was especially important for patients who were overdue to care that were most likely to benefit from the intervention and had not visited the clinics when consent could be obtained. The intervention posed no to minimal risks compared to existing routine care practices for which informed consent was previously obtained.

## Results

### Regular patients

**Fig. 1:**
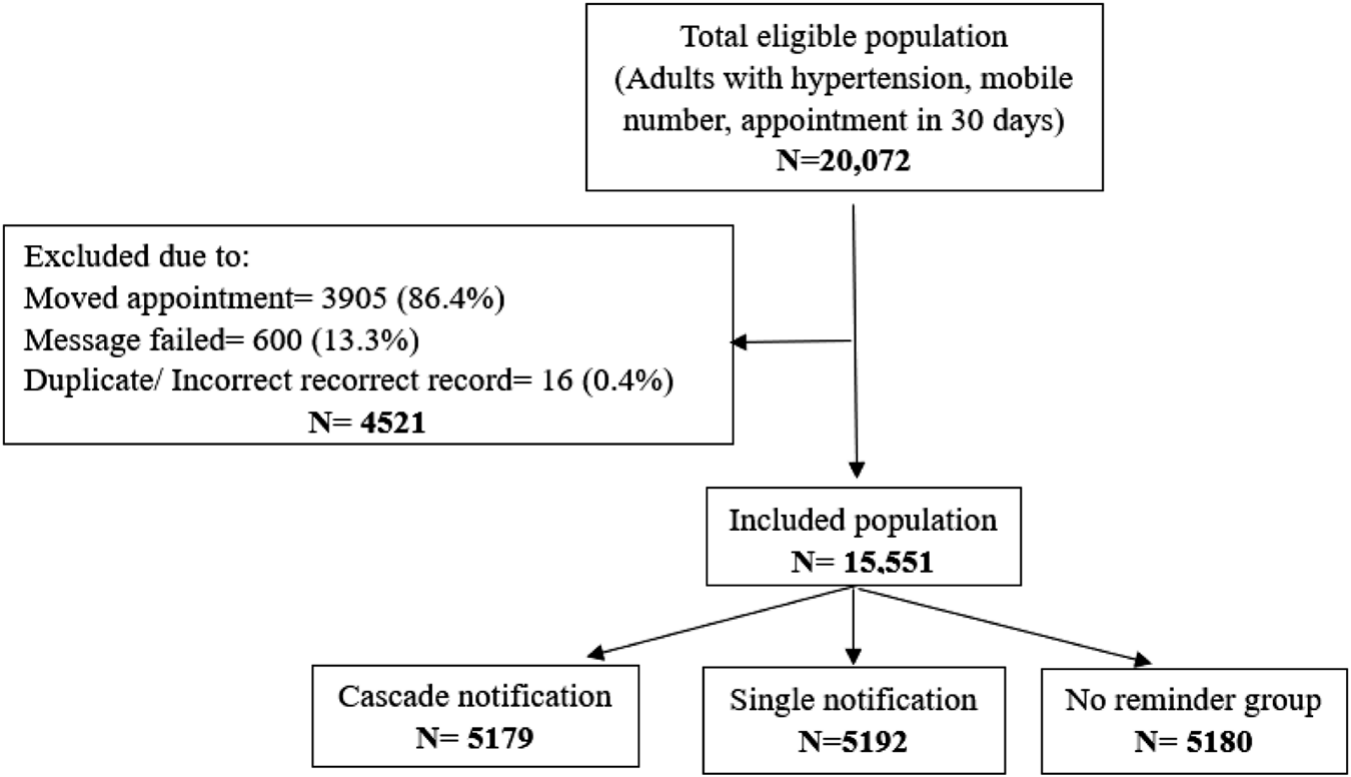
Population flow: regular patients.

During the study period, there were 20,072 eligible participants with a scheduled appointment and valid mobile telephone number who were enrolled as “regular patients” (Fig. 1). 4521 regular patients were excluded due to inadvertently moved appointments, failed messages, and duplicate or incorrect records.

At baseline, approximately one-third of the study population was male (35.3%), and the average age was 52.2 (SD 12.6) years (Table 2). The distribution of participant socio-demographic, clinical, and appointment-related characteristics was similar between arms and no significant differences were found in the regular patients (all *p* values > 0.05).

**Table 2:** Baseline characteristics of study participants.

Variables	Active patients (*n* = 15,551)				Overdue patients (*n* = 8099)		
	Single notification	Cascade notification	Control	p-value	Single notification	Control	*p*-value
Individuals (*n*)	5192	5179	5180		3958	4141	
Age (years), mean (SD)	52.1 (12.3)	52.2 (12.3)	52.4 (12.5)	0.294	52.4 (22.9)	52.1 (13.1)	0.286
Male	1809 (34.8)	1830 (35.3)	1788 (34.5)	0.695	1419 (35.9)	1491 (36.0)	0.879
History of diabetes	1669 (32.2)	1637 (31.6)	1598 (30.9)	0.361	940 (23.8)	908 (21.9)	0.051
History of kidney disease	12 (0.2)	9 (0.2)	10 (0.2)	0.801	26 (0.7)	10 (0.3)	0.005
History of heart attack	63 (1.2)	57 (1.1)	46 (0.9)	0.262	84 (2.1)	104 (2.5)	0.245
History of stroke	77 (1.5)	74 (1.4)	59 (1.1)	0.264	44 (1.1)	33 (0.8)	0.145
Baseline SBP (mmHg), mean (SD)	141.5 (20.4)	141.5 (20.0)	141.5 (20.2)	0.999	141.8 (20.9)	141.6 (20.2)	0.761
Baseline DBP (mmHg), mean (SD)	84.4 (12.0)	84.5 (11.8)	84.4 (11.9)	0.916	84.5 (12.5)	84.5 (12.3)	0.870
Time to last visit (days), Median (IQI)	29 (2)	29 (2)	29 (2)	0.760	70 (109)	74 (110)	0.117
History of missed visits	866 (16.7)	811 (15.7)	827 (16.0)	0.349	2358 (59.6)	2514 (60.7)	0.297
Duration in care (days), median (IQI)	71 (104)	68 (100)	71 (121)	0.760	131 (269)	129 (264)	0.530

*n* number of participants, *SBP* systolic blood pressure, *DBP* diastolic blood pressure, *IQI* interquartile interval.

[From overdue data, one transgender data was excluded from sex variables. Eighty-eight missing from time to last visit and blood pressure variables].

**Fig. 2:**
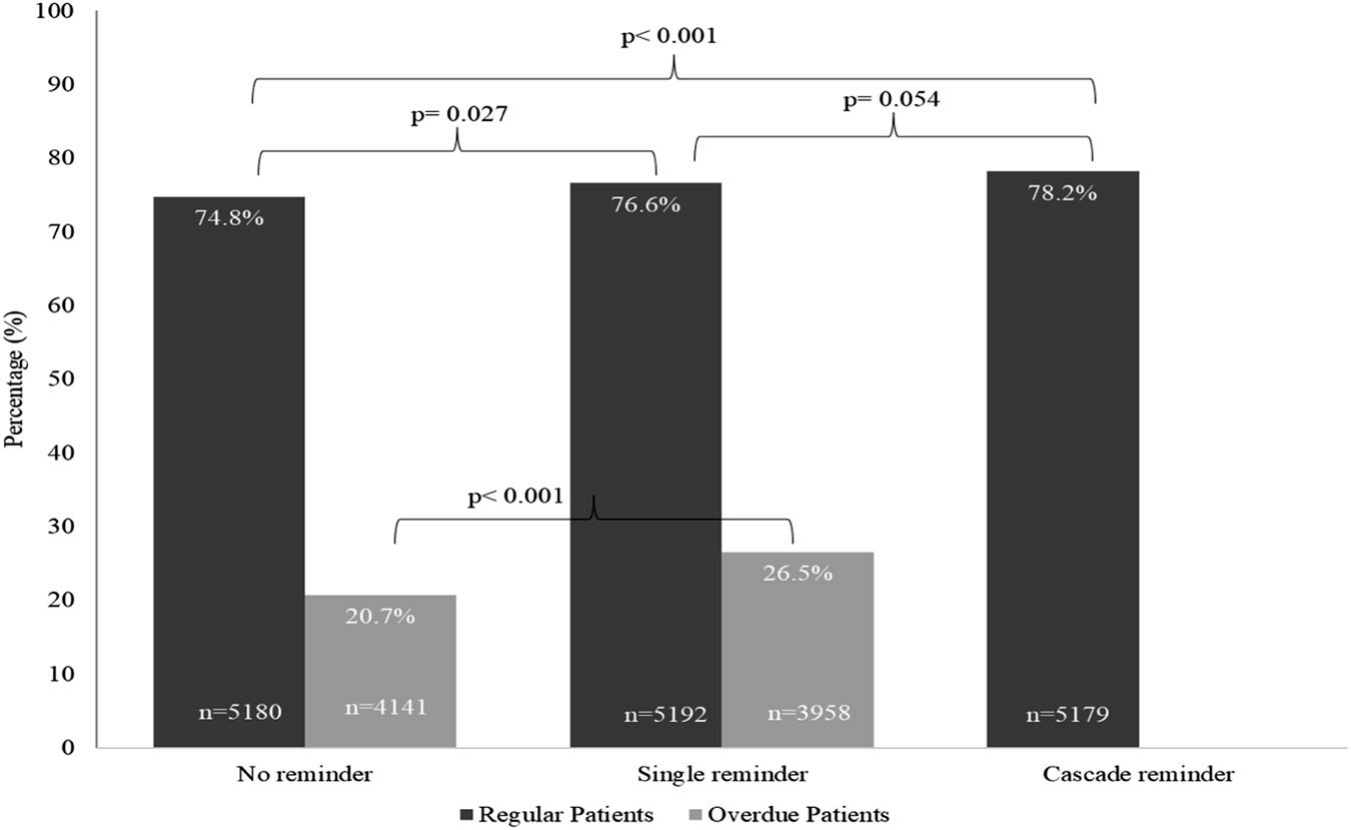
Visit attendance rates by text message reminder groups among regular and overdue patients.

Visit attendance was significantly more frequent in the cascade and single reminder groups compared to no reminder group (78.2% and 76.6% vs. 74.8%, *p* < 0.001 and 0.027, respectively) among regular patients (Fig. 2). The cascade message group had higher visit attendance than the single reminder group, although the difference was borderline significant (*p* = 0.05).

The results remained consistent in multivariable analysis with the no reminder group as a reference, with adjusted prevalence ratio (PR) of 1.04 (95% CI 1.02–1.06) for the cascade reminder group and 1.02 (95% CI 1.00–1.05) for the single reminder group (Table 3).

**Table 3:** Visit attendance by text message reminder group, unadjusted and adjusted analyses.

No reminder	Regular patients		Overdue patients	
	Unadjusted PR (95% CI)	*Adjusted PR (95% CI)	Unadjusted PR (95% CI)	*Adjusted PR (95% CI)
	Reference (1)	Reference (1)	Reference (1)	Reference (1)
Single reminder	1.03 (1.00–1.05)	1.02 (1.00–1.05)	1.28 (1.18–1.38)	1.23 (1.15–1.33)
Cascade reminder	1.05 (1.02–1.07)	1.04 (1.02–1.06)	—	—

*PR* prevalence ratio, *CI* confidence interval.

[* Adjusted for age, gender, geographic location. history of diabetes, kidney disease, heart attack, stroke, baseline-controlled BP, history of missed visits (defined as at least a three-month period without a visit in the past 12 months), time to last visit, and duration in care].

In secondary analysis including patients with failed messages, the follow-up visits rates though lower in all arms as expected, remained higher in the intervention groups compared to no reminder. Cascade and single message groups had higher visit attendance rates than no reminder for regular patients (77.3% and 75.7% vs. 74.8%, *p* = 0.002 and 0.238 respectively), although the difference between the single reminder group and no reminder group was not significant (Appendix 1). The results remained consistent in multivariable analysis with the no reminder group as a reference, with adjusted PR 1.03 (95% CI 1.01–1.05) for the cascade reminder group and 1.01 (95% CI 0.99–1.03) for the single reminder group (Appendix 2).

There were no significant differences in visit attendance by specific subgroups by age ( < 51 vs. ≥51 years), gender, history of missed visit, duration in care, time to last visit, and history of comorbidities (diabetes, kidney disease, heart attack, or stroke) (Appendix 3).

### Overdue patients

**Fig. 3:**
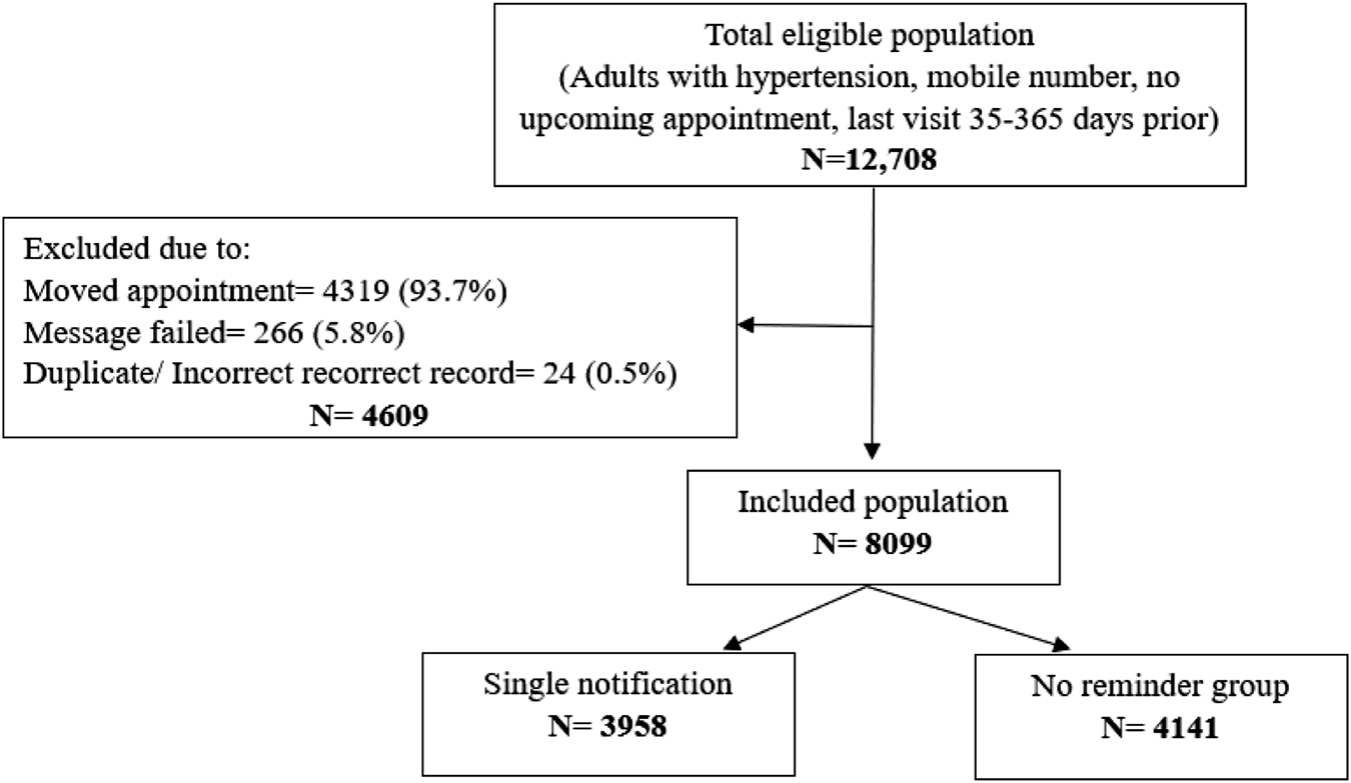
Population flow: overdue patients.

12,708 eligible participants with no upcoming appointment and last visit 35–365 days prior were enrolled as “overdue patients” (Fig. 3). 4609 overdue patients were excluded due to moved appointments, failed messages, and duplicate or incorrect records.

There were no significant differences in participant socio-demographic, clinical, and appointment-related characteristics between arms except for the history of kidney disease was more prevalent in the single reminder group vs. the no reminder group (*p*-value 0.005); however, the absolute difference was small (0.4%) (Table 2).

Visit attendance was higher in the single reminder group compared to the no reminder group among overdue patients (26.5% vs. 20.7%, *p* < 0.001) (Fig. 2). This difference of 5.8% unit in these two groups among overdue patients was even higher than the difference across the three groups in regular patients despite much lower visit rate ( < 30%) for overdue patients than for regular patients (75–80%). The results remained consistent in multivariable analysis with adjusted PR 1.23 (95% CI 1.15–1.33) for the single reminder group vs. the no reminder group (Table 3).

In secondary analysis including patients with failed messages, the visit attendance rates for overdue patients receiving a single message were also higher compared to no reminder (25.5% vs. 20.7%, *p* < 0.001) (Appendix 1). There were no significant differences in visit attendance by specific subgroups by age ( < 50 vs. ≥ 50 years), gender, history of missed visit, duration in care, time to last visit, and history of comorbidities (diabetes, kidney disease, heart attack, or stroke) (Appendix 3).

Visit attendance was higher for overdue patients whose last clinic was recent (i.e., last visit 35–90 days before study enrollment) compared to those who had a longer duration of non-attendance (last visit >180 days before study enrollment) (38.5% vs. 5.0% for the single reminder group, *p* < 0.001) (Appendix 4).

## Discussion

This study demonstrates that the use of text message reminders can improve clinic attendance for hypertension care in Bangladesh. Among regular patients, both cascade and single reminders improved visit attendance by 3.4% and 1.9% units respectively, where the baseline visit attendance rate (74.8%) was already high. The improvement was even more evident among patients overdue for care, where a single test message reminder showed a 5.8% unit improvement in visit attendance compared with the no reminder group (26.5% vs. 20.7%), corresponding to approximately 30% relative improvement. The patterns were consistent in sensitivity analyses and when we included patients with failed messages.

Several studies have shown that text messaging reminders can be effective and efficient for improving patients’ visit attendance [10, 12, 13, 16, 22–25]. However, most previous studies have been conducted in high-income countries, and few studies conducted in LMICs have focused on non-communicable diseases such as hypertension where loss to follow-up is highly prevalent. Additionally, little is known about the optimal message frequency and timing of text messages that results in improved visit attendance rates. To our knowledge, this is the first study to evaluate the impact of text reminders in improving attendance at primary healthcare facilities in Bangladesh. This study uniquely evaluates the use of a novel digital health platform, the Simple app, to facilitate automated text message reminders for hypertension and examine timing (before appointments vs. after missed appointments) and frequency (one reminder vs. three-message cascade).

In this study, among regular patients who were not overdue for care, the increase in return visit rates was modest, 3.4% unit (PR 1.04, 95% CI 1.02–1.06) with three-message cascade and 1.9% unit (PR 1.02, 95% CI 1.00–1.05) with single message compared to the no reminder group. Given the high baseline return visit rate (74.8%) for the no reminder group, there was limited room for improvement. However, despite the small increase of 2–3% unit in visit attendance rates, the return on investment at global scale may translate to millions of patients retained in care with low-cost, simple text messages.

The three-message cascade demonstrated a trend for improved return visit rates compared to the single message among regular patients (*p* = 0.05), suggesting that additional text messages may further nudge patients and result in even higher retention in care. However, further research is needed to characterize the optimal timing, frequency, and cost-effectiveness of SMS messages for retention in care.

Most prior studies have focused on reminders for prescheduled appointments sent before the appointment date but have not examined text messaging to patients already overdue for care. The larger effect seen in this study for overdue patients suggests focusing text reminders for patients after they miss a visit may be a particularly effective and efficient approach, especially in settings when baseline visit attendance is already high overall.

Additionally, text messages were more effective for recently overdue patients (i.e., last visit 35–90 days before study enrollment) compared to those who had a longer duration of non-attendance. This suggests that a single text message may not be a sufficient intervention for patients more distantly overdue who may require more intensive interventions, such as direct staff phone calls and/or community-based outreach. Other studies have shown that staff phone calls can be more effective in reducing non-attendance than text message reminders [22]. For overdue patients, a tiered strategy of outreach beginning with lower resource automated text message, followed by higher resource interventions including staff phone calls followed by community-based home outreach for those who do not return or do not have working phones warrants further investigation.

When compared to other methods, text message reminders allow large numbers of messages to be delivered simultaneously and automatically to reduce non-attendance at a relatively low cost with low labor intensity [23–25]. In this study, the cost per text message was 0.005 USD. The text message cost to return one regular patient to care was 0.27 USD and cost to return one overdue patient to care was 0.09 USD.

### Limitations

There are several limitations in this study that need to be considered. First, a sizeable proportion of eligible participants were excluded due to inadvertently moved appointments resulting from an intentional prompt in the mobile app user interface to encourage healthcare workers to schedule follow-up appointments. However, this user interface feature and resulting inadvertently moved appointments did not differ between study arms, and a significant increase in return visits was still present in intervention arms in the secondary analysis that included these patients (Appendix 2). Second, many people in rural Bangladesh do not own a mobile phone, particularly older adults, and they provide a relative’s mobile number when registering in the hypertension program. Therefore, although we were able to identify whether text messages were successfully transmitted to the listed number, we do not know whether the reminders were communicated to the patient in these cases. We are unable to assess the proportion of patients that provided a relative’s mobile number or whether there was a difference in visit attendance if the number provided belonged to the patient or someone else as this was not recorded in the digital tool. Furthermore, we did not assess literacy levels in this study nor the impact of text messaging on visit attendance compared to other adherence improvement methods. The results observed reflect real world conditions among the population in Bangladesh. Also, we did not formally assess cost-effectiveness or the effectiveness of text message reminders on medication adherence or BP control. However, prior research has demonstrated that increased visit frequency is strongly associated with BP control [26–28].

## Conclusion

Text message reminders can substantially increase the likelihood of returning for appointment visits for hypertension care in primary healthcare settings in Bangladesh, especially for patients overdue to care. The ease of sending bulk text messages at comparatively low cost provides a simple and efficient option to improve retention in care, a major barrier to effective hypertension care in LMICs. Future research should focus on testing optimal frequency and content of text messages for wider implementation in health services and to evaluate the effectiveness of text messaging on BP control.

## Summary

### What is known about this topic

- Hypertension is the leading cause of preventable death worldwide.
- Visit non-attendance is a common barrier to hypertension control in LMICs.
- Few studies have evaluated the effectiveness of text messages for improving visit attendance for hypertension in LMICs.

### What this study adds

- This study demonstrates that text message reminders can improve clinic attendance for hypertension care in Bangladesh.
- Text message reminders are an effective strategy for improving retention of patients in hypertension treatment in LMICs, especially for patients overdue to care.

## Supplementary information

Supplemental material

## Data Availability

De-identified data are available upon request by contacting the corresponding author.
