# Supplementary material for: Text messaging to improve retention in hypertension care in Bangladesh
Source: J Hum Hypertens. 2024 Aug 24;38(11):765–71. doi: 10.1038/s41371-024-00942-1 (PMC11543586; doi:10.1038/s41371-024-00942-1)
Supplement: Supplementary file 1 — Supplemental material [file 41371_2024_942_MOESM1_ESM.pdf]

Supplemental file:

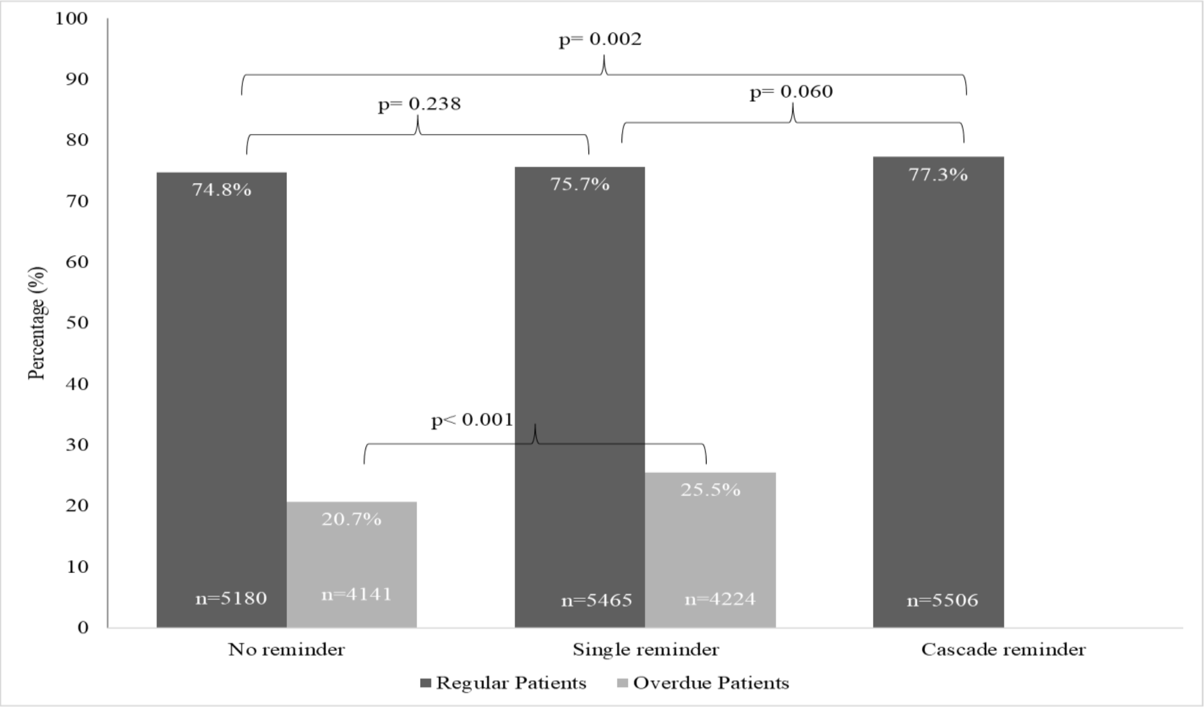

Appendix 1: Visit attendance by text message group including patients with failed messages

Appendix 2: Visit attendance by text message reminder group including patients with failed messages, unadjusted and adjusted analyses

	**Regular patients**		**Overdue patients**	
	Unadjusted PR (95% CI)	*Adjusted PR (95% CI)	Unadjusted PR (95% CI)	*Adjusted PR (95% CI)
No reminder	Reference (1)	Reference (1)	Reference (1)	Reference (1)
Single reminder	1.01 (0.99-1.04)	1.01 (0.99-1.03)	1.23 (1.14-1.33)	1.19 (1.11-1.28)
Cascade reminder	1.03 (1.01-1.06)	1.03 (1.01-1.05)	—	—

Abbreviations: PR, prevalence ratio; CI, confidence interval;

[* Adjusted for age, gender, geographic location. history of diabetes, kidney disease, heart attack, stroke, baseline-controlled BP, history of missed visits (defined as at least a three-month period without a visit in the past 12 months), time to last visit, and duration in care.]

Appendix 3: Prevalence ratios and 95% confidence intervals for visit attendance by prespecified groups

	Regular patients			Overdue patients	
	Cascade reminder vs. no reminder	Single reminder vs. no reminder	P-value for interaction	Single reminder vs. no reminder	P-value for interaction
As treated					
**Age**					
<median	1.05 (1.02-1.08)	1.04 (1.00-1.07)	0.658	0.92 (0.88-0.96)	0.650
≥ median	1.04 (1.01-1.07)	1.01 (0.98-1.04)		0.94 (0.91-0.96)	
**Sex**					
Male	1.06 (1.03-1.10)	1.04 (1.01-1.08)	0.283	0.93 (0.89-0.97)	0.598
Female	1.04 (1.01-1.06)	1.01 (0.99-1.04)		0.93 (0.90-0.95)	
**History of missed visit**					
Yes	1.02 (0.95-1.09)	1.01 (0.95-1.08)	0.424	0.95 (0.93-0.97)	0.348
No	1.05 (1.02-1.07)	1.03 (1.00-1.05)		0.90 (0.85-0.95)	
**Duration in care**					
<median	1.06 (1.03-1.09)	1.02 (0.99-1.06)	0.519	0.91 (0.87-0.94)	0.544
≥ median	1.03 (1.00-1.06)	1.03 (1.00-1.06)		0.95 (0.92-0.98)	
**Time to last visit**					
<median	1.07 (1.03-1.11)	1.03 (0.99-1.07)	0.452	0.90 (0.96-0.95)	0.096
≥ median	1.03 (1.00-1.06)	1.02 (1.00-1.05)		0.96 (0.94-0.98)	
**History of chronic diseases**					
Yes	1.02 (0.99-1.05)	1.00 (0.97-1.04)	0.474	0.95 (0.90-1.00)	0.146
No	1.06 (1.03-1.08)	1.03 (1.00-1.06)		0.92 (0.90-0.95)	

Appendix 4: Overdue patients, follow-up rate by days since last visit

	**Single Notification**		**No reminder**	
Days since last visit before enrollment	Number of participants with last visit before enrollment in each date range	Number (%) of participants with follow-up visit	Number of participants with last visit before enrollment in each date range	Number (%) of participants with follow-up visit
35-90 days	2378	915 (38.5)	2417	769 (31.8)
91-180 days	702	86 (12.3)	785	63 (8.0)
181-365 days	826	41 (5.0)	874	13 (1.5)
p value		<0.001		<0.001
